# Potential Genetic Markers Associated with Coloration in Duck: A Review

**DOI:** 10.3390/ijms262311460

**Published:** 2025-11-26

**Authors:** Muhammad Zahoor Khan, Qingshan Ma, Chunming Wang, Yongdong Peng, Mingxia Zhu, Changfa Wang

**Affiliations:** College of Agriculture and Biology, Liaocheng University, Liaocheng 252000, China; zahoorkhan@lcu.edu.cn (M.Z.K.);

**Keywords:** duck plumage coloration, melanogenesis, potential pigmentation genes

## Abstract

Plumage coloration in ducks (*Anas platyrhynchos*) represents a complex polygenic trait of significant economic and biological importance in commercial poultry production. This comprehensive review synthesizes current knowledge on the genetic mechanisms underlying feather coloration in domestic ducks, with particular emphasis on melanin biosynthesis pathways and their regulatory networks. We systematically analyzed recent advances including genome-wide association studies, RNA sequencing, whole-genome resequencing, and population genetics approaches that have identified key candidate genes controlling duck pigmentation patterns. The melanogenesis pathway emerges as the central regulatory network, with nine core genes (*MITF*, *MC1R*, *TYR*, *TYRP1*, *DCT*, *SOX10*, *KIT*, *EDNRB2*, and *MLANA*) consistently associated with plumage coloration across multiple duck populations. The *MITF* functions as the master regulator, coordinating expression of the enzymatic triad (*TYR*, *TYRP1*, *DCT*) responsible for melanin synthesis, while *MC1R* serves as the primary receptor controlling eumelanin versus pheomelanin production ratios. Epistatic interactions between *MITF* and *MC1R* demonstrate the complexity of color inheritance, with *MITF* exhibiting dominant effects over *MC1R* in determining white versus black plumage phenotypes. Functional enrichment analyses confirm these genes’ central roles in melanin biosynthetic processes and tyrosine metabolism pathways. Additionally, recent studies have revealed the importance of regulatory mechanisms, including epigenetic modifications and tissue-specific expression patterns, in modulating final coloration phenotypes. Understanding these genetic determinants provides valuable insights for selective breeding programs aimed at optimizing esthetic and economic traits in duck production. This review establishes a foundation for future research in avian pigmentation genetics and offers practical applications for improving breeding efficiency and product quality in the global duck industry.

## 1. Introduction

Duck (*Anas platyrhynchos*) represents one of the most economically important domesticated waterfowl species globally, valued for its meat production, egg laying capacity, and diverse byproducts [1,2,3]. China alone maintains over 32 indigenous duck breeds, each exhibiting distinctive phenotypic characteristics [4,5,6,7,8], particularly in feather coloration patterns that serve as valuable markers for breed identification and commercial applications [9,10]. Plumage coloration in avian species fulfills multiple critical biological functions beyond mere esthetic appeal, including thermoregulation, ultraviolet protection, cryptic camouflage, intraspecific communication, and sexual selection mechanisms [11,12,13,14,15]. These functional roles underscore the evolutionary significance of pigmentation systems and their continued importance in modern poultry breeding programs.

The remarkable diversity of coloration patterns observed across various poultry birds reflect the underlying complexity of pigmentation genetics, where multiple genes interact through intricate regulatory networks to produce the final phenotype [16,17,18]. Traditional breeding approaches have successfully selected for desired color traits, but the molecular mechanisms governing these characteristics have remained largely elusive until recent advances in genomic technologies [9]. Understanding the genetic architecture of duck coloration has become increasingly important as commercial breeding programs seek to optimize both esthetic qualities and associated production traits while maintaining genetic diversity within breeding populations [19,20,21].

Melanin represents the predominant pigment system responsible for the majority of coloration patterns observed in avian plumage, occurring as two principal forms: eumelanin, which produces black and brown colorations, and pheomelanin, responsible for red and yellow types [22,23,24,25,26]. The biosynthesis of these pigments follows well-characterized biochemical pathways involving sequential enzymatic reactions that convert the amino acid tyrosine into complex melanin polymers. However, the regulation of this process involves multiple levels of control, including transcriptional regulation, post-translational modifications, and tissue-specific expression patterns that collectively determine the final distribution and intensity of pigmentation [27,28,29].

Recent technological advances in genomics, including whole-genome sequencing, genome-wide association studies (GWAS), and RNA sequencing approaches, have revolutionized our understanding of the molecular basis of duck coloration [30,31]. These tools have enabled researchers to identify specific candidate genes, characterize their functional roles, and elucidate the complex regulatory networks that control pigmentation patterns [32,33,34]. Such knowledge not only advances our fundamental understanding of avian biology but also provides practical tools for improving breeding efficiency and developing marker-assisted selection programs.

The objective of this comprehensive review is to synthesize current knowledge regarding the genetic determinants of duck coloration, with particular emphasis on recent genomic discoveries and their functional significance. We examine the core genes involved in melanogenesis pathways, analyze their regulatory interactions, and discuss the practical implications for duck breeding programs. Additionally, we identify knowledge gaps and future research directions that will further advance our understanding of avian pigmentation genetics and its applications in commercial poultry production.

While this review focuses specifically on ducks, it is important to acknowledge that plumage coloration represents a trait of interest across the broader spectrum of poultry species, including chickens, quails, turkeys, and geese. The genetic architecture of coloration in ducks provides a foundation for understanding pigmentation mechanisms in other avian species, though comprehensive comparative reviews across all poultry birds remain an important area for future research. The narrow focus on ducks in this review reflects the current state of available genetic studies, and expanding this knowledge base to encompass other poultry species represents a critical priority for the field.

## 2. Candidate Genes Underlying Plumage Coloration in Ducks

Duck plumage coloration represents a complex polygenic trait influenced by multiple genes that regulate pigment cell development, melanin biosynthesis, and the spatial distribution of color. Through GWAS, transcriptomic screening, and targeted sequencing approaches, researchers have identified several promising candidate genes that appear to play crucial roles in determining the diverse color phenotypes observed across duck species and breeds.

### 2.1. Transcriptomic and Expression Profiling Studies

Recent advances in transcriptomic analyses utilizing high-throughput RNA sequencing technologies have significantly enhanced our understanding of the molecular mechanisms underlying melanin biosynthesis and plumage coloration in waterfowl species. Initial pioneering studies employing comparative transcriptomics identified *EDNRB2*, *TYR*, *KIT*, *EDNRB*, and *MC1R* as principal regulators of the melanogenic pathway, with their expression patterns demonstrating direct correlations with feather color phenotypes across multiple duck populations [35]. These genes encode critical components of the melanogenesis cascade: *TYR* serves as the rate-limiting enzyme catalyzing initial steps of melanin synthesis, while *MC1R* functions as the primary melanocortin receptor mediating hormonal signals that stimulate melanogenesis. Further validation came from integrated approaches combining RNA sequencing with GWAS in Youjiang goose populations, which revealed additional melanogenesis-related genes including *TYRP1*, *EDNRB2*, *DCT*, *TYR*, and *MLANA* that collectively regulate the melanogenic pathway and determine feather coloration phenotypes through coordinated expression [36]. These studies demonstrated that phenotypic variation in plumage coloration arises from the combinatorial action of multiple genes operating within interconnected regulatory networks, where TYRP1 and DCT function as critical enzymes in eumelanin synthesis, while MLANA (melan-A) plays structural roles in melanosome biogenesis and organization.

Parallel investigations focusing on skin pigmentation mechanisms consistently identified *TYR*, *ASIP*, *TYRP1*, and *KIT* as primary regulatory factors controlling dermal melanin deposition in ducks [37]. Building upon these findings, transcriptomic analysis examining melanin content variation in webbed feet revealed a striking positive correlation between pigmentation intensity and melanin concentration, with specimens exhibiting heavily pigmented webbed feet demonstrating maximal melanin content, while unpigmented feet showed undetectable melanin concentrations [38]. This phenotypic gradient corresponded precisely with differential expression patterns of critical melanin biosynthesis genes, including *TYRP1*, *PMEL*, *DCT*, *TYR*, *OCA2*, *MC1R*, *RAB38*, *WNT16*, *CAMK2A*, and *MLANA* in Magang goose populations [38]. Importantly, darkly pigmented tissues exhibited significantly upregulated expression of pro-melanogenic genes, with some genes showing 2–5 fold increases in transcript abundance compared to unpigmented tissues, where PMEL encodes the premelanosome protein essential for melanosome structural integrity, while OCA2 regulates melanosomal pH and influences melanin polymer characteristics.

Expanding this molecular framework, recent integrative genomic and transcriptomic analyses have identified twelve candidate genes (*MITF*, *MC1R*, *TYR*, *TYRP1*, *ABCB6*, *DGKI*, *GPRC5B*, *HMX1*, *STS*, *ADGRA1*, *PRKAR2B*, and *HOXB9*) significantly associated with melanin biosynthesis and plumage trait determination in Matahu duck populations through combined quantitative trait locus (QTL) mapping and expression profiling approaches [9]. This comprehensive gene panel encompasses core melanogenic enzymes, upstream transcriptional regulators (*MITF*, *HMX1*, *HOXB9*), membrane transporters (*ABCB6*), and signaling molecules, indicating that plumage pigmentation is controlled by a multilayered regulatory architecture. Investigations into dorsoventral color variation patterns in Light Brown Mottling ducks, utilizing RNA-seq analysis of embryonic skin tissues from dorsal and ventral regions, revealed that key melanogenesis-related genes (*ASIP*, *OCA2*, *MLANA*, *MC1R*, *TYR*, and *TYRP1*) showed statistically significant differential expression between dorsal and ventral anatomical regions [27]. Specifically, *ASIP* (agouti signaling protein) likely plays a decisive role in determining dorsoventral plumage patterns through its function as an endogenous antagonist of *MC1R* signaling, competitively inhibiting melanocortin receptor activity and promoting pheomelanin production over eumelanin synthesis.

Comprehensive transcriptomic investigations examining sex-specific differences have successfully elucidated sexual dimorphism in avian pigmentation patterns through coordinated regulation of melanogenesis genes. Research on Hungarian white goose goslings revealed that female goslings consistently exhibit darker dorsal down coloration compared to males, with melanin content measurements showing 1.5–2 fold higher levels in females [39]. This sex-linked dimorphism is orchestrated by coordinated regulation of melanogenesis-related genes including *MC1R*, *TYR*, *TYRP1*, *DCT*, and *MITF*, with *MC1R* and *MITF* showing substantially higher expression levels in female feather follicles, directly correlating with increased melanin synthesis rates. Similarly dramatic sexual dimorphism is observed in mallard duck feather coloration, characterized by males displaying brilliant iridescent green head feathers while females exhibit dull brown coloration. This phenotypic difference results from substantially increased melanosome deposition in male head feather barbules, arranged in a distinctive hexagonal lattice structure that generates the characteristic iridescent appearance through structural coloration mechanisms [40]. Comparative transcriptome analysis identified *TYR* and *TYRP1* showing significantly elevated expression levels in male head feather follicles, with *TYRP1* exhibiting an extraordinary 256-fold increase compared to female head follicles and a 32-fold elevation compared to male back follicles [40]. Complementary research examining *TYRP1* and *ASIP* expression patterns in Holdobaggy goslings further elucidated sex-linked coloration differences, where female goslings demonstrated significantly higher *TYRP1* expression levels and correspondingly elevated melanin content, correlating with darker gray and black down coloration [23]. Conversely, male goslings exhibited elevated *ASIP* expression, associated with lighter, buff-colored plumage coloration, demonstrating that these genes exert opposing regulatory effects through antagonistic interactions between pro- and anti-melanogenic factors.

Moreover, transcriptomic profiling has revealed unexpected involvement of four homeobox genes functioning as developmental transcription factors and two glutathione metabolism-related genes, specifically ChaC glutathione-specific gamma-glutamylcyclotransferase 1 (*CHAC1*) and glutathione peroxidase 3 (*GPX3*), in black feather formation and melanin deposition [41]. The identification of glutathione metabolism genes is particularly intriguing, as glutathione plays critical roles in redox homeostasis and can influence melanin biosynthesis through its effects on reactive oxygen species levels within melanocytes, suggesting that transforming growth factor-β (TGF-β) signaling pathways may also contribute to the complex regulatory network governing avian pigmentation patterns.

In a complementary line of investigation, studies in Muscovy ducks, a domestic breed derived from a distinct species compared to common ducks, employed quantitative real-time PCR (qPCR) analysis with careful normalization to reference genes to identify *FNDC1* (fibronectin type III domain containing 1) and *ADAMTS12* (a disintegrin and metalloproteinase with thrombospondin motifs 12) as genes showing significantly elevated expression associated with white plumage color phenotypes [42], suggesting that these genes may suppress melanogenesis or promote melanocyte dysfunction. Conversely, *MYOT* (myotilin) and *MB* (myoglobin) were identified as genes showing enhanced expression linked to black plumage color [43], although the direct mechanistic connections between these muscle-related proteins and melanin biosynthesis remain to be fully elucidated and warrant further investigation. Collectively, these expression-based studies, encompassing multiple species, breeds, tissue types, and analytical methodologies, demonstrate the remarkable power and utility of transcriptomic approaches in identifying candidate genes, revealing their tissue-specific and developmental expression patterns, and elucidating their regulatory roles and functional contributions in avian pigmentation, thereby establishing a comprehensive molecular framework for understanding plumage color determination in waterfowl species.

### 2.2. Genetic Association and Variant Analysis

The advent of GWAS and high-throughput sequencing technologies has fundamentally transformed our understanding of the genetic architecture underlying duck plumage variation. These approaches have evolved from identifying individual candidate genes to revealing complex regulatory networks that orchestrate pigmentation patterns through coordinated molecular mechanisms.

Multiple independent studies have converged on *MC1R* and *MITF* as central regulators of duck pigmentation, with consistent associations identified across diverse populations and analytical approaches [29,44,45,46,47,48,49,50,51]. In *MC1R*, two non-synonymous single nucleotide polymorphisms (SNPs) in the *MC1R* gene (c.52G>A and c.376G>A on ZJU1.0 assembly) demonstrate robust associations with black plumage phenotypes, likely enhancing receptor activity and downstream eumelanin synthesis [44]. Concurrently, MITF variants consistently associate with white plumage across Chinese Crested, Cherry Valley, and Putian duck populations, with three key SNPs (chr13:15411658A>G, chr13:15412570T>C, and chr13:15412592C>G on ZJU1.0 assembly) potentially disrupting this master transcriptional regulator’s function [44,47,48,51]. Furthermore, the pleiotropic effects of these genes extend beyond feather pigmentation to beak coloration, as demonstrated in Mallard and Pekin populations where MITF and POU2F3 jointly regulate melanin deposition patterns [49].

Building upon these foundational discoveries, recent high-resolution GWAS analyses have revealed that pigmentation complexity involves sophisticated developmental programs extending well beyond classical melanogenic pathways. Notably, the identification of *SOX10*, a neural crest transcription factor functioning upstream of *MITF*, alongside *VWA5A* in coordinated regulatory mechanisms highlights the importance of melanocyte developmental cascades in determining adult coloration [50,52]. Particularly significant is the discovery that specific *SOX10* variants (Chr1.g.54065419C>T and g.54070844C>T) simultaneously influence both pigmentation and reproductive performance, suggesting evolutionary constraints that link color phenotypes to fitness-related traits [52]. These finding challenges traditional views of pigmentation as purely cosmetic and reveals underlying genetic correlations with organismal function.

In parallel investigations, the genetic landscape of duck pigmentation has expanded dramatically with the identification of genes involved in cellular metabolism, protein processing, and ion transport. Studies in Brown Tsaiya and Ji’an Red ducks have implicated *GMDS* (fucose biosynthesis), *ODC1* (polyamine metabolism), and *PDIA6* (protein folding) in red plumage determination, while *ASIP* maintains its established role in pheomelanin production [53,54]. These findings suggest that pigmentation outcomes depend not only on melanogenic enzyme activity but also on cellular metabolic state and protein quality control mechanisms. Similarly, the identification of calcium channel subunits (*CACNA1I*, *CACNA2D4*) and G-protein signaling components (*GNAO1*) in male-specific green head coloration indicates that pigmentation involves calcium-dependent regulatory pathways affecting melanosome function [55].

Moreover, comprehensive analyses across duck breeds reveal both conserved mechanisms and breed-specific genetic signatures that contribute to phenotypic diversity. In Tianfu Nonghua ducks, an extensive panel including *WNT3A*, *DOCK1*, *RAB1A*, *ALDH1A3*, and ion transporters (*SLC24A1*) regulates regional pigmentation patterns, while complementary studies in Nonghua duck populations identify *STK4*, *CCN5*, and *YWHAB* as determinants of discrete spotted patterns [56,57]. The involvement of vesicular trafficking genes (*RAB1A*, *AP3B1*, *VAMP7*), chromatin modifiers (*SMARCA2*, *SETD6*), and metabolic regulators across multiple breeds suggests that pigmentation phenotypes emerge through integrated cellular processes affecting melanocyte development, melanosome transport, and gene expression regulation [55,56,57,58,59].

Extending these observations to broader phylogenetic contexts, comparative genomic analyses across waterfowl species demonstrate remarkable conservation of core pigmentation mechanisms while revealing species-specific regulatory variations. The identification of *TYR*, *SLC45A2*, *SLC7A11*, and *PWWP2A* in swan coloration, alongside the extensive gene panel (*KITLG*, *KIT*, *TYRO3*, *AP3B1*) identified in geese, establishes that fundamental melanogenic pathways have been maintained throughout waterfowl evolution [59,60]. Consequently, this conservation-variation pattern suggests that pigmentation diversity arises primarily through regulatory evolution affecting gene expression patterns and epistatic interactions rather than structural protein diversification. The consistent identification of trafficking components (*AP3B1*), transcriptional regulators (*MITF*, *SOX10*), and metabolic genes across species indicates that successful pigmentation requires coordination of multiple cellular processes, establishing a framework for understanding both the mechanistic basis and evolutionary constraints shaping waterfowl coloration diversity.

### 2.3. Population Genomics Approaches

Advancements in population genomics have revolutionized the identification of candidate genes under selection through comparative whole-genome analyses and fixation index (Fst) statistics, which measure genetic differentiation between populations with distinct phenotypic traits [61,62,63]. These approaches provide unique insights that complement functional studies by revealing genomic regions experiencing selection pressure during domestication and breed development, accomplished through detection of selective sweeps characterized by reduced genetic diversity, elevated linkage disequilibrium, and shifts in allele frequency spectra.

Population-based analyses have consistently identified a core set of melanogenic genes across multiple waterfowl species and breeds, providing robust evidence for their central roles in pigmentation determination. Whole-genome resequencing of Korean native duck populations and Fst-based selective sweep analysis in Jianchang ducks converged on *DCT*, *KIT*, *TYR*, and *MC1R* as major candidates showing significant allelic differentiation between colored and white phenotypic variants [61,62]. Similarly, fixation index testing in Liancheng white duck populations revealed *KIT*, *MITF*, and additional regulatory genes under selection for white feather coloration [63]. The consistent identification of these genes across independent populations and analytical frameworks indicates strong artificial selection pressure during breed development, where *DCT* and *TYR* function as core melanogenic enzymes, *KIT* regulates melanocyte development and migration, and *MC1R* mediates melanogenic signaling cascades.

Importantly, population genomics has expanded the genetic landscape beyond classical melanogenesis pathways by identifying novel candidate genes that influence pigmentation through diverse cellular mechanisms. Comparative analyses of Chinese duck breeds revealed *SPATA2*, *EIF2S2*, *PLIN3*, *ATP1B1*, and *CCDC80* as additional loci contributing to distinctive coloration phenotypes, suggesting that optimal melanogenesis requires coordination with protein translation machinery, lipid metabolism, and cellular ion homeostasis [5]. Furthermore, the identification of *CLOCK*, a core circadian rhythm regulator, in Liancheng white duck populations introduces an intriguing temporal dimension to pigmentation control, potentially linking circadian biology to the timing of melanin synthesis during feather development [63]. These findings indicate that population-level selection has acted not only on core pigmentation genes but also on regulatory networks that optimize cellular conditions for melanogenesis.

Extending these observations across waterfowl taxa, population differentiation analyses in geese have reinforced the importance of conserved pigmentation mechanisms while revealing species-specific genetic signatures. Fst analysis identified *KIT* as exhibiting significant population differentiation between white and gray plumage variants in geese, while whole-genome resequencing in Huoyan geese revealed *TYRP1* and *GDA* as genes associated with feather color and skin pigmentation [64,65]. Concurrently, Sanger sequencing in Wugangtong goose populations identified *EDNRB2* and *MLANA* as contributors to plumage color variation [66]. The identification of *GDA*, an enzyme involved in purine metabolism, represents a particularly novel finding suggesting that nucleotide metabolism may influence pigmentation through mechanisms affecting cellular energy status or redox balance in melanocytes.

Collectively, these population-based genomic approaches provide crucial evolutionary context that complements transcriptomic and association mapping studies by revealing which genes have been targets of artificial selection during breed development. Unlike transcriptomics, which identifies differentially expressed genes between phenotypes, or GWAS, which detects statistical associations in segregating populations, population genomics reveals the historical selective forces shaping genetic variation. The convergent identification of genes such as *MITF*, *MC1R*, *KIT*, and *EDNRB2* across transcriptomic, GWAS, and population genomic analyses provides particularly strong evidence for their central roles in waterfowl pigmentation [5,47,48,61,62,63,64,65,66,67]. This multi-faceted approach reveals that duck plumage coloration emerges from coordinated action of multiple genetic networks encompassing core melanogenic enzymes, developmental regulators, and novel cellular pathways involving circadian regulation, metabolic processes, and ion homeostasis. The integration of these diverse molecular mechanisms through population-level selection has generated the remarkable phenotypic diversity observed in domestic waterfowl breeds, establishing a comprehensive framework for understanding both the mechanistic basis and evolutionary dynamics of avian pigmentation. For comprehensive reference, the potential genes associated with various coloration phenotypes in ducks are summarized in Table 1.

## 3. Molecular Architecture and Regulatory Mechanisms of Melanogenesis in Duck Plumage Development

### 3.1. Overview of Core Melanogenesis Genes

**Figure 1:**
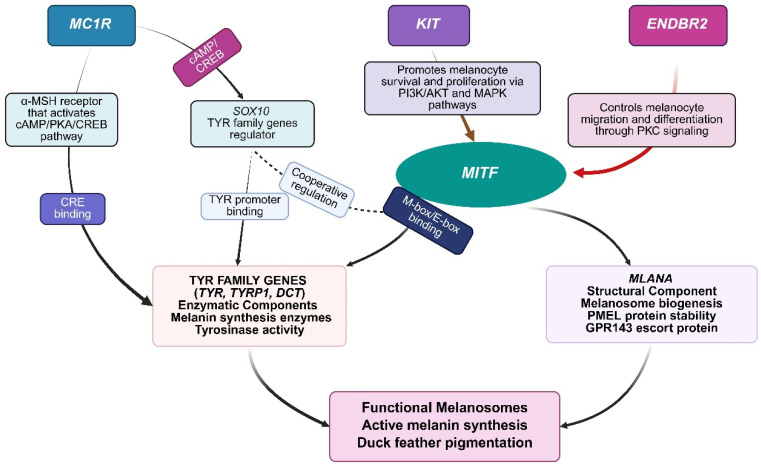
Duck melanogenesis regulatory network, showing how multiple upstream signals (*MC1R*, *KIT*, *EDNRB2*) coordinate through specialized transcription factors (*SOX10*, *MITF*) to control both enzymatic (TYR family genes) and structural (*MLANA*) components needed for functional melanosomes and feather pigmentation. The figure illustrates that *MC1R* as an α-MSH receptor that activates cAMP/PKA/CREB signaling, providing both direct regulation of TYR genes through CREB binding to CRE elements and indirect control via *SOX10* activation. *SOX10* functions as a specialized transcription factor specifically targeting TYR family genes (*TYR*, *TYRP1*, *DCT*) and works cooperatively with *MITF* for optimal gene expression. This creates a four-input convergence system where *MC1R*, *KIT*, and *EDNRB2* signals coordinate through dual transcriptional control—*SOX10* specializing in *TYR* family regulation while *MITF* serves as the master regulator for both enzymatic (TYR family) and structural (*MLANA*) components. The network demonstrates how multiple upstream pathways (cAMP/PKA from *MC1R*, PI3K/MAPK from *KIT*, and Protein kinase C (PKC) from *EDNRB2*) integrate through specialized transcription factors to ensure coordinated expression of all components necessary for functional melanosome biogenesis and active melanin synthesis in duck feather pigmentation, with particular relevance to understanding how genetic variations in these pathways contribute to diverse plumage patterns. This figure relies on unverified, preliminary information and should be interpreted cautiously, as the illustrated relationships have not been independently validated.

Based on comprehensive literature review across diverse research methodologies including GWAS, RNA-seq, whole-genome resequencing, and FST analysis, nine genes consistently emerge as central regulators of duck pigmentation: *MITF*, *MC1R*, *TYR*, *TYRP1*, *DCT*, *SOX10*, *KIT*, *EDNRB2*, and *MLANA* [35,36,68,69]. These genes represent distinct functional categories within the melanogenesis pathway: the core enzymatic triad (*TYR*, *TYRP1*, *DCT*) directly catalyzes melanin synthesis; the primary receptor *MC1R* controls eumelanin versus pheomelanin production; transcriptional regulators *MITF* and *SOX10* serve as master controllers of melanocyte development and specification; and developmental signaling genes (*KIT*, *EDNRB2*, *MLANA*) mediate melanocyte migration, survival, and melanosome function. The melanogenesis regulatory cascade operates hierarchically: *MC1R* activation by α-melanocyte stimulating hormone triggers cyclic AMP elevation and CREB phosphorylation, which binds to CRE elements in the *TYR* promoter upregulating tyrosinase expression, while *MITF* activation through phosphorylation stimulates transcription of *TYR*, *TYRP1*, and *DCT*, with melanin synthesis occurring within specialized melanosomes that subsequently transfer to keratinocytes. These genes have demonstrated reproducible associations with pigmentation phenotypes across multiple duck and chicken populations documented in the majority of published investigations reviewed in Section 2, establishing them as reliable targets for genetic analysis and breeding applications. The information regarding these nine selected genes from reported studies is provided in Table 2, and the duck melanogenesis regulatory gene network (*TYR* family genes and key regulators including *MITF*, *MC1R*, *KIT*, *EDNRB2*, *SOX10*) is presented in Figure 1.

### 3.2. Master Transcriptional Regulation Through MITF

The *MITF* gene represents a pivotal regulatory node in melanocyte biology, functioning as a basic helix-loop-helix leucine zipper transcription factor that coordinates melanocyte development, survival, and functional capacity throughout vertebrate lineages [51]. Operating within a complex regulatory framework, *MITF* integrates signals from diverse upstream pathways while orchestrating downstream melanogenic responses [33]. The molecular mechanism involves specific DNA-protein interactions at E-box regulatory sequences within promoter regions of target genes, including the critical enzymatic components *TYR*, *TYRP1*, and *TYRP2/DCT* [9,30,70], whose coordinated activation represents the biochemical foundation of melanin production.

Within duck feather follicles, *MITF* demonstrates remarkable cell-type specificity through differential isoform expression. The melanocyte-specific isoform *MITF-M* exhibits exclusive expression within melanocytes of black feather bulbs, while being entirely suppressed in melanocytes from white feather bulbs [51]. This binary expression pattern has been documented across numerous duck populations, including Putian, Liancheng, Peking, Shanma, Wendeng black duck, and various Asian indigenous lines, positioning *MITF-M* as a fundamental determinant of black-versus-white plumage dichotomy [46,51].

Multiple regulatory layers govern *MITF* expression through both genetic variation and epigenetic modifications. Genome-wide association analyses have identified eight polymorphic positions within the proximal 2000 bp upstream of the *MITF-M* transcription start site, each demonstrating significant influence on melanin biosynthesis by modulating transcription factor recruitment efficiency [54]. Within Asian duck breeds, particular genetic variants have emerged, including synonymous substitutions in exon 1 and a 14-bp insertion-deletion polymorphism in intron 7, both showing robust statistical associations with plumage coloration diversity [71]. Pan et al. [70] established that the polymorphism ASM874695v1:10:g.17814522T>A within *MITF* correlates significantly with black beak pigmentation, while independent investigations documented *MITF* SNP associations with white feather manifestation in Kaiya × Liancheng crossbred populations [72]. Epigenetic mechanisms provide an additional dimension of regulation, with CpG island methylation within the *MITF* promoter demonstrating inverse correlation with *MITF-M* transcript levels [73]. Comparative analyses in quail species revealed CpG methylation levels of 22% in Korean quail versus 30% in Beijing white quail, directly correlating with darker versus white plumage pigmentation [74]. From an evolutionary perspective, Zhou et al. [29] identified a novel intronic insertion within *MITF* in Pekin ducks that likely disrupts normal splicing patterns, resulting in the white down feather phenotype characteristic of domesticated birds.

### 3.3. Melanocortin Receptor Signaling and Pigment Type Determination

Operating in concert with *MITF*, the melanocortin 1 receptor (*MC1R*) gene encodes a seven-transmembrane domain G-protein-coupled receptor that serves as the primary melanogenic signal transducer in domestic duck populations [75]. Ligand binding by α-melanocyte stimulating hormone (α-MSH) triggers *MC1R*-mediated adenylate cyclase activation, resulting in elevated intracellular cyclic AMP concentrations [76,77]. This second messenger cascade subsequently activates protein kinase A, which phosphorylates the transcription factor *CREB* to enhance *MITF* transcription alongside other melanogenic genes, ultimately driving eumelanin production—the dark brown to black pigment class responsible for darker feather phenotypes [26,78]. Conversely, when *MC1R* signaling is attenuated through competitive antagonist binding by molecules such as *ASIP*, the melanogenic pathway shifts toward pheomelanin synthesis, producing lighter, reddish-yellow pigmentation [79].

Systematic genetic characterization across Asian duck breeds has revealed extensive *MC1R* polymorphism underlying plumage variation. Sultana et al. [71] cataloged twelve *MC1R* polymorphisms distributed across seven Asian duck breeds, with five coding region SNPs producing amino acid substitutions. Among these variants, four nonsynonymous substitutions demonstrate significant phenotypic associations: c.52A>G (p.Lys18Glu), c.376A>G (p.Ile126Val), c.409G>A (p.Ala137Thr), and c.649C>T (p.Arg217Cys) [70]. Tu et al. [79] and Yu et al. [80] confirmed that c.52G>A and c.376G>A substitutions strongly associate with the extended black variant, with these alleles appearing to confer enhanced *MC1R* signaling capacity, promoting constitutive eumelanin deposition across duck plumage [20,79,80,81,82,83]. Beyond coding sequence variation, Liu et al. [34] identified four novel regulatory region SNPs demonstrating strong black plumage associations, indicating that melanistic phenotypes result from both receptor functional alterations and expression level modulation.

### 3.4. Enzymatic Machinery and Supporting Regulatory Components

Following *MITF* activation through *MC1R* signaling or alternative pathways, direct transcriptional activation of *TYR*, *TYRP1*, and *TYRP2/DCT* genes occurs through *MITF* binding at their respective promoters. These genes encode the enzymatic core of melanin biosynthesis: *TYR* catalyzes the rate-limiting oxidation of tyrosine to DOPA and subsequently to dopaquinone, *TYRP1* channels intermediates toward eumelanin synthesis while stabilizing tyrosinase catalytic activity, and *TYRP2/DCT* mediates dopachrome tautomerization [71]. The orchestrated expression of these three enzymes under *MITF* control determines both melanin type and quantity, with prior investigations documenting associations between *TYR* family genes and feather coloration in chicken [68,84], quail [85], and duck populations [38]. Temporal analysis of beak pigmentation reveals that the immediate post-hatch period (0–7 days) is characterized by *EDNRB* signaling and *MITF* expression driving early melanosome maturation, while sustained *TYR*, *TYRP1*, and *DCT* expression during weeks 4–6 enables continued melanin synthesis essential for maintaining stable black-beak phenotypes [70].

Beyond these core melanogenic enzymes, several supporting components are essential for proper pigmentation. The *KIT* proto-oncogene encodes a receptor tyrosine kinase indispensable for melanocyte survival, proliferation, and migration, with *c-Kit* transcript levels in black feather bulbs exceeding those in white feather bulbs by approximately 10-fold [46]. *KIT* activation initiates MAPK and PI3K/AKT signaling cascades that promote melanocyte survival while phosphorylating and activating *MITF*, establishing a reinforcing feedback circuit that stabilizes the melanogenic cellular state. The *EDNRB2* gene plays an essential role in melanocyte migration and spatial distribution, thereby controlling pigmentation pattern formation. Using GWAS analysis in 225 ducks, two significant *EDNRB2* variants (Chr4:10,180,939 T>C and Chr4:10,190,671 A>T) were identified that are predicted to disrupt transcription factor binding sites, accounting for 49.5% and 32.9% of the spot size variation on the dorsal and ventral surfaces, respectively [28]. Additionally, the *PMEL* is essential for melanosome structural maturation by forming intralumenal fibrillar scaffolds that provide templates for melanin polymer deposition, with two candidate SNPs identified in Liancheng ducks demonstrating significant association with plumage coloration traits [33].

### 3.5. Epistatic Interactions and Hierarchical Gene Networks

Avian pigmentation complexity is further exemplified by epistatic interactions wherein one gene masks or modifies another’s phenotypic effects. Compelling evidence demonstrates epistatic interactions between *MC1R* and *MITF* genes: certain ducks exhibiting white plumage carried *MC1R* variants c.52G>A and c.376G>A, which typically produce black and spotted plumage phenotypes [44]. These observations suggest *MITF* functions as an upstream regulatory gene relative to *MC1R* in controlling coloration patterns, as *MITF* loss-of-function can completely mask *MC1R* gain-of-function effects [44,69]. This epistatic relationship reflects the hierarchical organization of melanogenic pathways, wherein *MITF* serves as the master transcriptional regulator activating all downstream melanogenic genes. Without functional *MITF*, even constitutively active *MC1R* cannot generate pigment because melanogenic enzymes (*TYR*, *TYRP1*, *DCT*) remain untranscribed, effectively positioning *MITF* downstream of multiple signaling inputs but upstream of the entire melanogenic machinery. This integrated understanding of the hierarchical genetic network—encompassing upstream signaling through *MC1R* and *KIT*, master regulation by *MITF*, enzymatic machinery of *TYR* family genes, structural components like *PMEL*, and spatial patterning by *EDNRB2*—provides a comprehensive framework for understanding pigmentation traits in duck breeding programs [44].

### 3.6. Evolutionary Conservation and Cross-Species Validation

Comparative genomic analyses reveal substantial evolutionary conservation of pigmentation genes between ducks and other avian species, suggesting shared molecular mechanisms underlying coloration across avian taxa [59,68,84,85]. Several candidate genes identified in ducks, including *MC1R*, *MITF*, *TYR*, and *SOX10*, have been independently associated with plumage coloration in chickens, demonstrating remarkable functional conservation despite millions of years of evolutionary divergence [68,84]. For instance, *MC1R* variants control black plumage in both chickens and ducks [20,79,80,81,82,83], while *MITF* serves as a master regulator of melanogenesis across multiple bird species [59]. Cross-species validations in quail further support these conserved mechanisms, with *MITF* expression patterns correlating with pigmentation phenotypes [74,85]. The conservation of core pigmentation machinery across avian species suggests evolutionary constraints on these pathways, while genetic variation within these networks provides targets for selective breeding programs. These findings strengthen confidence in the functional importance of these genes and provide valuable insights for future marker development in understudied poultry species where direct genetic mapping may be more challenging.

## 4. Functional Pathway Analysis and Gene Network Integration

To characterize the biological functions and regulatory networks of nine pigmentation-related genes (*KIT*, *MITF*, *DCT*, *SOX10*, *TYRP1*, *TYR*, *MC1R*, *MLANA*, and *EDNRB2*), which were selected based on their consistent identification across multiple genomic studies and their well-established roles in melanogenesis pathways, we performed GO enrichment and KEGG pathway analyses using ShinyGO v0.80 [86] and DAVID v2024 [87]. The *Anas platyrhynchos* (Mallard duck) genome was used as the background reference, with significance thresholds set at *p* < 0.05 for GO terms and FDR < 0.05 for KEGG pathways. Functional enrichment analysis revealed significant involvement in melanin biosynthetic processes, pigmentation, melanocyte differentiation, and pigment cell development, with secondary enrichment in neural crest cell development, cell fate commitment, and tyrosine metabolism (Table 3) [72,88,89,90,91].

KEGG pathway analysis identified Melanogenesis (apla04916) [88,89,90,91] as the most significantly enriched pathway, with seven genes (*MITF*, *TYR*, *TYRP1*, *DCT*, *MC1R*, *KIT*, *SOX10*) directly participating [72]. Within this network, *MC1R* initiates cAMP signaling, *MITF* functions as the master transcriptional regulator integrating multiple pathways (cAMP, Wnt, p38 MAPK), *TYR*/*TYRP1*/*DCT* constitute the enzymatic triad for melanin synthesis, *KIT* mediates melanocyte proliferation and survival, and *SOX10* regulates neural crest-derived melanocyte development. The Tyrosine metabolism pathway (apla00350) emerged as the second most significant pathway (*p* < 0.01), where *TYR* catalyzes rate-limiting steps (tyrosine → DOPA → DOPAquinone), *TYRP1* converts DHI to DHICA, and *DCT* facilitates DOPAchrome conversion to DHICA.

This coordinated network demonstrates that duck color variation primarily results from quantitative and qualitative changes in melanin production, with the *TYR*-*TYRP1*-*DCT* complex determining eumelanin-to-pheomelanin ratios. The conservation of these genes across the melanogenesis pathway suggests evolutionary constraint on core pigmentation machinery and provides targets for selective breeding. The biological processes and pathways implicated in duck pigmentation that are modulated by the nine selected genes are summarized in Table 3 and Table 4. These consolidated data provide additional evidence supporting the functional involvement of these genes in regulating avian coloration.

## 5. Conclusions and Future Perspectives

This comprehensive review identifies nine core genes (*MITF, MC1R, TYR, TYRP1, DCT, SOX10, KIT, EDNRB2,* and *MLANA*) as fundamental regulators of duck coloration through coordinated melanogenesis pathways. The enzymatic triad (*TYR*, *TYRP1*, *DCT*) directly catalyzes melanin synthesis, while transcriptional regulators (*MITF*, *SOX10*) control expression patterns and developmental signaling genes (*KIT*, *EDNRB2*, *MC1R, MLANA*) manage melanocyte function. Complex epistatic interactions among these genes create the diverse plumage phenotypes observed across duck breeds, providing robust targets for marker-assisted selection.

The genetic markers identified in this review hold significant potential for implementation in genomic selection programs, a breeding strategy that has revolutionized genetic improvement in major livestock species. While coloration may not be considered as economically critical as production traits such as growth rate or disease resistance, it remains an important selection criterion in certain breeding contexts. Plumage coloration serves as a breed-defining characteristic essential for maintaining breed purity and meeting market preferences in specialty duck markets. Moreover, coloration genes may exhibit pleiotropic effects on physiologically relevant traits, such as immune function or stress response, making them relevant for comprehensive genetic evaluation. Integration of coloration markers into multi-trait genomic selection indices could enable simultaneous improvement of appearance traits (including plumage coloration, skin color, and breed-typical phenotypes) and production traits while maintaining acceptable rates of genetic gain across the breeding objective. Although direct genetic correlations between plumage coloration and production traits (e.g., growth rate, egg production) have not been extensively characterized in ducks, studies in chickens have reported low to moderate correlations between pigmentation genes and body weight, suggesting potential pleiotropic effects that warrant further investigation in waterfowl.

While substantial progress has been made, several important considerations merit attention for future research. The majority of current studies have focused on candidate gene approaches and population-level associations, with limited functional validation through direct experimental manipulation. Functional characterization through CRISPR-Cas9 genome editing can directly validate the causal role of candidate variants, while single-cell RNA sequencing can elucidate cell-type-specific regulatory mechanisms during feather development. Additionally, the interactions between identified genes and environmental factors, including nutrition and lighting conditions, remain poorly characterized and warrant systematic investigation. The development of cost-effective SNP panels targeting validated coloration markers would enable routine genotyping in breeding programs, facilitating marker-assisted selection even in resource-limited settings. These genomic tools will require validation across diverse duck populations and breeding contexts to ensure broad applicability. Integration of these markers into existing genetic evaluation systems, combined with multi-gene predictive models, would allow breeders to make informed decisions about coloration while simultaneously selecting for economically important production traits. Expanding genomic analyses to rare breeds and wild populations will enhance understanding of pigmentation evolution, ultimately advancing breeding efficiency and enhancing both the esthetic and commercial value of duck populations.

## Figures and Tables

**Table 1:** Summary of genes associated with duck color phenotypes.

Breed	Potential Genes	Screening Method	Color Associated Phenotypic Traits	Reference
Longsheng duck	*EDNRB2*, *MITF*, *SPATA2*, *EIF2S2*, *PLIN3*, *ATP1B1*, *CCDC80*	Comparative genomics (FST)	Distinctive coloration phenotype	[5]
Matahu duck	*MITF*, *MC1R*, *TYR*, *TYRP1*, *ABCB6*, *DGKI*, *GPRC5B*, *HMX1*, *STS*, *ADGRA1*, *PRKAR2B*, *HOXB9*	QTL mapping and expression profiling	Melanin biosynthesis, plumage trait determination	[9]
Holdobaggy goose	*TYRP1*, *ASIP*	Expression profiling	Sex-linked dorsal plumage patterns	[23]
Light Brown Mottling duck	*ASIP*, *OCA2*, *MLANA*, *MC1R*, *TYR*, *TYRP1*	RNA-seq (dorsal vs. ventral embryonic skin)	Dorsoventral color variation	[27]
Multiple duck populations	*EDNRB2*, *TYR*, *KIT*, *EDNRB*, *MC1R*	RNA-seq (transcriptomic screening)	Melanogenic pathway regulation, feather color correlation	[35]
Youjiang goose	*TYRP1*, *EDNRB2*, *DCT*, *TYR*, *MLANA*	Integrated RNA-seq and GWAS	Melanogenic pathway, feather coloration	[36]
Duck skin tissues	*TYR*, *ASIP*, *TYRP1*, *KIT*	Transcriptomic analysis	Skin pigmentation control	[37]
Magang goose	*TYRP1*, *PMEL*, *DCT*, *TYR*, *OCA2*, *MC1R*, *RAB38*, *WNT16*, *CAMK2A*, *MLANA*	RNA-seq of webbed feet	Melanin content variation, dose-dependent pigmentation	[38]
Hungarian white goose	*MC1R*, *TYR*, *TYRP1*, *DCT*, *MITF*	Transcriptomic analysis	Sex-specific pigmentation, sexual dimorphism in goslings	[39]
Duck (general)	*CHAC1*, *GPX3*	Transcriptomic profiling	Black feather formation, melanin deposition	[41]
Muscovy duck	*FNDC1* and *ADAMTS12*	quantitative real-time PCR (qPCR)	White color	[42]
Muscovy duck	*MYOT* and *MB*	qPCR	Black plumage color	[43]
Multiple duck breeds	*MC1R* (c.52G>A, c.376G>A) *MITF* (chr13:15411658A>G, chr13:15412570T>C, chr13:15412592C>G)	GWAS and variant analysis	Black plumage (*MC1R*); white plumage (*MITF*)	[44]
Multiple duck populations	*MITF*	Whole-genome sequencing	Plumage color across diverse populations	[29,45,46]
Chinese Crested duck and Cherry Valley duck	*MITF* and *EDNRB2*	GWAS	Associated with black and white color plumage	[47]
Chinese Crested duck and Cherry Valley duck	*MITF* and *EDNRB2*	GWAS	Regulate melanin synthesis and variation in beak color	[48]
Mallards and Pekin ducks	*MITF* and *POU2F3*	GWAS	Melanin deposition in duck beak	[49]
Multiple duck breeds	*VWA5A*, *MITF*, *SOX10*	GWAS with increased marker density	Plumage color coordination	[50]
Putian black ducks	*MITF*	GWAS	Associated with regulation of black and white plumage coloration	[51]
White Kaiya and white Liancheng ducks	*SOX10*(g.54065419C>T and g.54070844C>T)	Gene sequencing	Associated with white feathers coloration	[52]
Brown Tsaiya and Ji’an Red duck	*GMDS*, *ODC1*, *PDIA6*	GWAS	Red plumage and feather color variation	[53]
Chaohu and Ji’an red ducks	*ASIP* and *LOC101797494*	Whole-genome sequencing	Pigmentation and plumage color	[54]
Multiple duck breeds	*CACNA1I*, *WDR59*, *GNAO1*, *CACNA2D4*, *LOC101800026*, *SYNPO2*, *MXI1*	GWAS	Green head traits, *TYRP1* regulation	[55]
Tianfu Nonghua ducks	*WNT3A*, *DOCK1*, *RAB1A*, *ALDH1A3*, *DPP8*, *HACD3*, *INTS14*, *SLC24A1*, *DENND4A*, *PRKG1*, *SETD6*, *RALYL*, *ZNF704*	GWAS	Associated with color pigment on the dorsal and ventral feathers of the ducks Regulate pigmentation	[56]
Nonghua ducks	*STK4*, *CCN5*, and *YWHAB*	GWAS	Regulate melanin-related pathways or pigment deposition, Associated black spot on feathers and	[57]
Longyan Shan-ma ducks	*ZNF106*, *SLC7A5*, *BANP ZNF106 STARD9*, *SLC7A5*, *BANP*, *LOC101798015*, and *IPMK*	GWAS	Involved in pigmentation and follicle development	[58]
Geese	*KITLG*, *MITF*, *TYRO3*, *KIT*, *AP3B1*, *SMARCA2*, *ROR2*, *CSNK1G3*, *CCDC112*, *VAMP7*, *SLC16A2*, *RLIM*, *KIAA2022*, *ST8SIA4*, *TRPM6*, *TICAM2*	GWAS	Regulate feather color in geese	[59]
Swan populations	*TYR*, *SLC45A2*, *SLC7A11*, *PWWP2A*	Comparative genomics	Melanin production, plumage coloration	[60]
Korean native duck	*DCT*, *KIT*, *TYR*, *ADCY9*	Whole-genome resequencing (FST)	Pigmentation pattern differentiation	[61]
Jianchang duck	*MITF* and *MC1R*	F_ST_ analysis	Hemp and white feathers	[62]
Liancheng white duck	*KIT*, *CLOCK*, *MITF*, *CEBPA*	Fixation index (F_ST_) test	White color feather Regulate melanin pathway	[63]
Geese	*KIT*	F_ST_ analysis	White/gray plumage color	[64]
Huoyan geese	*TYRP1* and *GDA*	Whole-genome resequencing	Feathers color phenotypes and skin pigmentation	[65]
Wugangtong goose	*EDNRB2* and *MLANA*	Sanger sequencing	Plumage colors	[66]
Shitou geese	*TYR*, *TYRP1*, *EDNRB2*, *MLANA*, *SOX10*, *SLC45A2*, *GPR143*, *TRPM1*, *OCA2*, *ASIP*, *KIT*, *SLC24A5*	RNA-Seq	White feather follicles	[67]

**Table 2:** Information regarding selected coloration-linked genes in duck.

Symbol	Ensembl Gene ID	Species	Chr	Position (Mbp)	nExons
*SOX10*	ENSAPLG00000015217	Duck	1	53.368843	3
*DCT*	ENSAPLG00000013837	Duck	1	163.367528	10
*TYR*	ENSAPLG00000012676	Duck	1	175.170443	4
*KIT*	ENSAPLG00000004054	Duck	4	44.96999	21
*MC1R*	ENSAPLG00000000850	Duck	12	20.222742	1
*MITF*	ENSAPLG00000011965	Duck	13	15.388146	11
*MLANA*	ENSAPLG00000003877	Duck	Z	29.800872	4
*TYRP1*	ENSAPLG00000013453	Duck	Z	34.236291	8

**Table 3:** Biological processes related to coloration in duck.

Term	Genes	*p*-Value	Description
GO:0043473	*KIT*, *MITF*, *DCT*, *SOX10*, *TYRP1*, *TYR*	7.85 × 10^−14^	Pigmentation
GO:0048066	*KIT*, *MITF*, *DCT*, *SOX10*, *TYRP1*	4.26 × 10^−13^	Developmental pigmentation
GO:0030318	*KIT*, *MITF*, *SOX10*, *TYRP1*	6.72 × 10^−11^	Melanocyte differentiation
GO:0042438	*DCT*, *TYRP1*, *TYR*	6.14 × 10^−9^	Melanin biosynthetic process
GO:0002052	*DCT*, *SOX10*	1.33 × 10^−5^	Positive regulation of neuroblast proliferation

**Table 4:** KEGG signaling pathways related to pigmentation and coloration in duck.

Category	Term	*p*-Value	Genes
KEGG_PATHWAY	apla04916: Melanogenesis	1.10 × 10^−9^	*MC1R*, *DCT*, *TYRP1*, *KIT*, *MITF*, *TYR*
KEGG_PATHWAY	apla00350: Tyrosine metabolism	3.36 × 10^−4^	*DCT*, *TYRP1*, *TYR*

## Data Availability

No new data were created or analyzed in this study. Data sharing is not applicable to this article.

## Abbreviations

ABCB6	ATP binding cassette subfamily B member 6
ADAMTS12	ADAM metallopeptidase with thrombospondin type 1 motif 12
ADCY9	Adenylyl cyclase 9
ADGRA1	Adhesion G protein-coupled receptor A1
ALDH1A3	Aldehyde dehydrogenase 1 family member A3
AP3B1	Adaptor related protein complex 3 subunit beta 1
ASIP	Agouti signaling protein
ATP1B1	ATPase Na+/K+ transporting subunit beta 1
BANP	BTG3 associated nuclear protein
CACNA1I	Calcium voltage-gated channel subunit alpha1 I
CACNA2D4	Calcium voltage-gated channel auxiliary subunit alpha2delta 4
CAMK2A	Calcium/calmodulin-dependent protein kinase II alpha
CCDC112	Coiled-coil domain containing 112
CCDC80	Coiled-coil domain containing 80
CCN5	Cellular communication network factor 5
CEBPA	CCAAT enhancer binding protein alpha
CHAC1	ChaC glutathione-specific gamma-glutamylcyclotransferase 1
CLOCK	Clock circadian regulator
cMYB	MYB proto-oncogene
CREB	cAMP response-element binding protein
CSNK1G3	Casein kinase 1 gamma 3
DCT	Dopachrome tautomerase (also known as TYRP2)
DENND4A	DENN domain containing 4A
DGKI	Diacylglycerol kinase iota
DOCK1	Dedicator of cytokinesis 1
DPP8	Dipeptidyl peptidase 8
EDNRB2	Endothelin receptor type B2
EIF2S2	Eukaryotic translation initiation factor 2 subunit beta
FNDC1	Fibronectin type III domain containing 1
GDA	Guanine deaminase
GMDS	GDP-mannose 4,6-dehydratase
GNAO1	G protein subunit alpha o1
GPR143	G protein-coupled receptor 143
GPRC5B	G protein-coupled receptor class C group 5 member B
GPX3	Glutathione peroxidase 3
HACD3	3-hydroxyacyl-CoA dehydratase 3
HMX1	H6 family homeobox 1
HOXB9	Homeobox B9
INTS14	Integrator complex subunit 14
IPMK	Inositol polyphosphate multikinase
KIAA2022	KIAA2022
KIT	KIT proto-oncogene
KITLG	KIT ligand (also known as Stem cell factor)
LOC101798015	Uncharacterized gene
LOC101800026	Uncharacterized gene
MB	Myoglobin
MC1R	Melanocortin 1 receptor
MITF	Microphthalmia-associated transcription factor
MLANA	Melan-A (melanoma antigen recognized by T-cells 1)
MXI1	MAX interactor 1
MYOT	Myotilin
OCA2	Oculocutaneous albinism II
ODC1	Ornithine decarboxylase 1
PDIA6	Protein disulfide isomerase family A member 6
PLIN3	Perilipin 3
PMEL	Premelanosome protein
POU2F3	POU class 2 homeobox 3
PRKAR2B	Protein kinase cAMP-dependent type II regulatory subunit beta
PRKG1	Protein kinase cGMP-dependent 1
PWWP2A	PWWP domain containing 2A
RAB1A	RAB1A, member RAS oncogene family
RAB38	RAB38, member RAS oncogene family
RALYL	RALY RNA binding protein like
RLIM	Ring finger protein, LIM domain interacting
ROR2	Receptor tyrosine kinase like orphan receptor 2
SETD6	SET domain containing 6
SLC16A2	Solute carrier family 16 member 2
SLC24A1	Solute carrier family 24 member 1
SLC24A5	Solute carrier family 24 member 5
SLC45A2	Solute carrier family 45 member 2
SLC7A11	Solute carrier family 7 member 11
SLC7A5	Solute carrier family 7 member 5
SMARCA2	SWI/SNF related matrix associated actin dependent regulator of chromatin subfamily A member 2
SOX10	SRY-box transcription factor 10
SPATA2	Spermatogenesis associated 2
ST8SIA4	ST8 alpha-N-acetyl-neuraminide alpha-2,8-sialyltransferase 4
STARD9	StAR related lipid transfer domain containing 9
STK4	Serine/threonine kinase 4
STS	Steroid sulfatase
SYNPO2	Synaptopodin 2
TICAM2	TIR domain containing adaptor molecule 2
TRPM1	Transient receptor potential cation channel subfamily M member 1
TRPM6	Transient receptor potential cation channel subfamily M member 6
TYR	Tyrosinase
TYRO3	TYRO3 protein tyrosine kinase
TYRP1	Tyrosinase-related protein 1
VAMP7	Vesicle associated membrane protein 7
VWA5A	Von Willebrand factor A domain containing 5A
WDR59	WD repeat domain 59
WNT16	Wnt family member 16
WNT3A	Wnt family member 3A
XBP1	X-box binding protein 1
YWHAB	Tyrosine 3-monooxygenase/tryptophan 5-monooxygenase activation protein beta
ZNF106	Zinc finger protein 106
ZNF704	Zinc finger protein 704
